# Co-Designing a Digital Platform to Support a Culturally Adapted Family Intervention (CaFI:Digital) for Psychosis Among People of Sub-Saharan African and Caribbean Descent: Agile Co-Design Approach

**DOI:** 10.2196/73246

**Published:** 2026-03-25

**Authors:** Pauline Whelan, Henna Lemetyinen, Helen Morley, Heidi Tranter, Simon Foster, Dawn Edge

**Affiliations:** 1 Division of Informatics, Imaging and Data Science, School of Health Sciences, University of Manchester, Manchester, United Kingdom; 2 GM.Digital Research Unit, Research and Innovation, Greater Manchester Mental Health NHS Foundation Trust, Greater Manchester, United Kingdom; 3 Perinatal Mental Health and Parenting Research Unit, Research and Innovation, Greater Manchester Mental Health NHS Foundation Trust, Greater Manchester, United Kingdom; 4 Division of Psychology and Mental Health, Faculty of Biology, Medicine and Health, University of Manchester, Oxford Rd, Manchester, M13 9PL, United Kingdom, 44 161 306 6000; 5 Manchester Centre for Health Psychology, Division of Psychology & Mental Health, University of Manchester, Manchester, United Kingdom; 6 Equality, Diversity and Inclusion Research Unit, Research and Innovation, Greater Manchester Mental Health NHS Foundation Trust, Greater Manchester, United Kingdom; 7 NIHR Manchester Biomedical Research Centre, Manchester, United Kingdom

**Keywords:** health inequalities, digital mental health, psychosis, mental illness, accessibility, co-design methods, family therapy, culturally adapted

## Abstract

**Background:**

People of sub-Saharan African and Caribbean descent are significantly more likely to be diagnosed with psychotic disorders than other ethnic groups in the United Kingdom. The National Institute for Health and Care Excellence in the United Kingdom recommends family therapy as a clinically effective treatment for the management of psychosis. The National Institute for Health and Care Excellence also recommends that family interventions should be culturally informed to meet the needs of an increasingly ethnically diverse population. People from minoritized backgrounds are rarely offered family therapy; however, the rise in digital mental health worldwide offers unique opportunities to support culturally informed approaches at scale and at a low cost.

**Objective:**

The overarching aim of culturally adapted family intervention (CaFI):Digital was to help address inequalities in the provision of mental health care for people of sub-Saharan African and Caribbean descent, including those of Mixed heritage. A digital platform, CaFI:Digital, was built to support delivery of a CaFI. The purpose of developing CaFI:Digital was to provide an accessible, user-friendly, and engaging website for service users, their families, and therapists as an alternative or adjunct to in-person therapy.

**Methods:**

We used an iterative Agile co-design approach to develop a user-friendly and inclusive website. Co-design workshops (n=2), semistructured interviews (n=2), and collaborative research team meetings (n=3) were used to capture and prioritize end-user feedback on the clinician- and service-user–facing components of the platform. The software was developed using Agile sprints, with each sprint lasting 3 weeks, allowing feedback to be integrated rapidly and revised software prototypes to be shared with end users for review, revision, and approval.

**Results:**

Key software requirements, such as accessibility and diverse content, were identified in the co-design activities and were implemented to maximize accessibility and usability of the website. Following software development, we successfully beta-tested the software with our target end user population of service users and therapists to ensure it was defect-free and ready for use.

**Conclusions:**

A digital platform to support delivery of CaFI for psychosis was rapidly developed through a series of co-design activities. To our knowledge, this is the first bespoke digital therapy platform that has been co-designed with and for people of sub-Saharan African and Caribbean descent who experience psychosis. This is important given the disproportionate rates of diagnosis and lack of access to psychological therapies experienced by this population.

## Introduction

In the United Kingdom, people of sub-Saharan African and Caribbean descent, including those of Mixed heritage, have significantly elevated risk of diagnosis with schizophrenia and related psychoses compared with White British peers [1,2]. Black and Mixed heritage service users attribute this increased risk of psychosis, at least partly, to the buildup of stressors directly linked to ethnic minority status in general and racism in particular [1]. Disparities in diagnostic rates are compounded by suboptimal care and negative experiences of services, which are characterized by high rates of involuntary hospitalization and inferior access to evidence-based treatments [1,3]. There is strong evidence for the cost- and clinical-effectiveness of psychological or “talking” therapies such as family intervention for psychosis [4,5]. However, despite National Institute for Health and Care Excellence (NICE) recommendations, such treatments are rarely offered to racialized populations. Neither are they culturally informed [6,7]. Moreover, while there are studies highlighting ethnically based inequalities in access to psychological therapies, we are not aware of any robustly evaluated digital psychological interventions specifically designed for Black populations diagnosed with schizophrenia or psychosis. This population is therefore underserved both digitally and in access to evidence-based care.

A culturally adapted family intervention (CaFI) was co-designed with people of sub-Saharan African and Caribbean descent diagnosed with schizophrenia and associated psychoses and other key stakeholders [8]. The CaFI trial was designed to provide in-person culturally appropriate group psychological therapy for people who have experienced longstanding disparities in mental health care and outcomes [9,10], including rarely being offered talking treatments [9,11]. Online therapies may increase engagement and uptake among a patient population with a history of fear and mistrust of engaging with statutory mental health services [12]. In the United Kingdom, NICE recommends culturally sensitive approaches for the treatment of psychosis, as well as family intervention therapy [13]. Funding from the National Institute of Health and Social Care Research in the United Kingdom [14] supported the original development of CaFI [15] and subsequently funded a randomized controlled trial, the “CaFI trial,” to investigate the effect on relapse of CaFI compared to usual care among people of sub-Saharan African and Caribbean descent diagnosed with psychosis in the United Kingdom [16].

The CaFI trial was originally planned to commence in April 2020 but was postponed due to the COVID-19 pandemic. As evidence emerged of higher rates of COVID-19 infections and mortality among Caribbean, African, and other ethnic minority populations in the United Kingdom [17], it became apparent that delivering in-person therapy was neither feasible nor ethical. In our work to develop CaFI, community members cited examples of Black people’s exploitation in research, most notably the use of Henrietta Lacks’ cells [18] and the Tuskegee syphilis study [19]. Thus, the development of a digital alternative was explored with former service users and community members and determined to be a potentially viable approach to improving the likelihood of engagement and participation in the study [20].

CaFI:Digital (a subcomponent of the CaFI trial: ISRCTN12622538) was created to support the delivery of online group therapy, enabling the study to continue while face-to-face consultations were not possible. The purpose of developing CaFI:Digital was to provide an accessible, user-friendly, and engaging website for service users, their families, and therapists as an alternative or adjunct to in-person therapy. CaFI:Digital is a web-based platform that provides access to a specially designed, cocreated therapy manual and resources to support therapeutic engagement. It was anticipated that the co-design of CaFI:Digital would ensure the platform was useful for service users, even after in-person therapy could resume. The CaFI trial was underpinned by the ethical imperative to improve the care of a population that has been underserved. To address trust and power issues, CaFI:Digital adopted a co-design approach that placed service users and their carers at the heart of the digital development process. The aim of this preliminary paper is to outline the specific co-design steps and work undertaken with key partners and stakeholders to develop CaFI:Digital. This includes strategies to ensure its usability and acceptability to end users. This paper will not outline methods or results of the CaFI trial, as this is still ongoing.

## Methods

### Ethical Considerations

Ethical approval was obtained for the CaFI trial from the Health Research Authority in the United Kingdom (19/NW/0385; IRAS ID 254857, approved July 24, 2019). Participants were provided with an information sheet and consent form ahead of the workshops to ensure informed consent was obtained. Participants were reimbursed for their time during workshops in line with National Institute for Health Research rates. Identifiable information was removed from workshop feedback to ensure the data was anonymized, in line with privacy and confidentiality guidance.

### Study Setting, Participants, Recruitment, and Sample

Participants were recruited through the participating National Health Service (NHS) community mental health teams and collaborating third-sector organizations. Participants were approached by email via established networks. The sampling method was purposive-opportunistic, and all participants who responded to email approaches were invited to attend the workshops. Service user and carer respondents were included if they were individuals of sub-Saharan African and Caribbean descent and either had lived experience of psychosis or were a carer or family member of an individual who had experienced psychosis. Therapist respondents were included if they were trained health care professionals who had clinical experience of working with people with psychosis. The mixed stakeholder group comprised a combination of people with lived experience of psychosis and health care professionals. Demographic data of participants in each research activity were not recorded consistently; this is a noted limitation of this project. This was a patient and public involvement and engagement activity rather than a research participant activity, with feedback collected from the groups and iteratively updated in the designs. The website was co-designed using an iterative, user-centered process over a 6-month period. Three workshops were held consisting of (1) service users and carers (n=7), (2) therapists (n=4), and (3) a mixed stakeholder workshop. Two individual semistructured interviews were also conducted separately with therapists during the design process. Research team meetings (n=3) were also held to capture and prioritize end-user feedback on the clinician- and service-user–facing components of the website. To ensure the team had time to engage with all participants in the virtual workshops, the maximum number for each session was set to 8 members. Staff time for the therapist workshops was difficult to obtain; therefore, the therapist group workshop was supplemented with additional individual interviews. The mixed stakeholder group was designed to accommodate participants who were unable (because of time or other resource constraints) to attend the single stakeholder groups. The sample size was guided by the concept of “information power” [21], which emphasizes the quality and comprehensiveness of data over the quantity of participants. Rather than focusing solely on data saturation, researchers are encouraged to aim for data sufficiency, particularly when studies have narrow aims, allowing for smaller yet richer samples [22].

Workshops were conducted using Microsoft Teams and lasted between 60 and 90 minutes. The workshops followed a similar structure, starting with an overview of the project followed by a discussion of key features, requirements, and priorities for CaFI:Digital. Design prototypes were presented at each workshop to allow participants to see what the website would look like and enable the participants to provide feedback on each iteration of the designs. The prototype user interface was designed in Adobe XD [23], and interactivity was added using Marvel [24]. Usability testing was conducted using Maze [25].

### Co-Design and Agile Approaches

A co-design approach was combined with Agile software methodologies [26] to support collaboration between people with lived experience, software developers, researchers, and designers. The co-design and user research approach followed the UK Government Service Design Guidelines across the 3 setup stages: Discovery, Alpha, and Beta [27]. Workshops were facilitated by the project manager, the project technical team lead, and a UX designer. In the Discovery phase workshop, service users and carers (n=7) were asked to comment on key requirements and features for the website (see Table 1).

**Table 1.** Workshop 1 input design changes—service user and carer workshop (Discovery phase).

Feedback item	Design/software modification	Project solution
(1) Accessibility		
Ensure navigation of the site is as simple and clear as possible for service users.	Navigation of the site simplified and updated.	Therapists trained by workshop facilitators to email or text a simple web link to service users to navigate to the required webpage.
Ensure the website works well for people with visual impairments. The option to make the text larger through the browser is really helpful, as many people have visual impairments.	Website reviewed to be Web Content Accessibility Guidelines 2.1 [18] compliant. Larger-than-usual font size used. Color contrast used to ensure readability.	Training by workshop facilitators on how to increase font size through the browser provided to the research team and therapists.
Provide a “Help Page” to guide users on how to engage with the content and materials.	Development of a “Help Page”	A “Help Page” included on the website to guide users on how to engage with the content and materials.
Some service users will not have access to technology to view CaFI^a^:Digital.	No action required.	Tablets purchased and provided to service users and families where needed. The study purchased mobile data plans to ensure the website could be accessed.
Some service users will not have the skills to engage with a website.	An emphasis on simplicity of design and implementation was maintained throughout.	Volunteer digital navigators at the lead NHS^b^ trust joined the project team to support service users with basic literacy skills, enabling them to access and use the website with support if they wished to do so. Volunteer digital navigators receive appropriate NHS training to support service users in developing and using digital skills.
The therapist would need to explain upfront what the website does.	No action required.	Training provided to therapists to ensure they pass on key information to service users before the website is recommended for use.
(2) Diverse content		
There should be more diversity in the images of people displayed across the website; specifically, a greater range of skin tones should be included.	No action required.	The images were updated to include a broader range of people representing diverse ethnic backgrounds (see Figure 1).
(3) Positive feedback		
Participants felt the website was very exciting and noted it was the first time such a resource has been developed for this population.	No action required.	No action required.
Participants felt the site is visually appealing.	No action required.	No action required.
Participants felt the site looked very simple to use.	No action required.	No action required.

a CaFI: culturally adapted family intervention.

b NHS: National Health Service.

The Alpha phase involved 3 stages; after each stage, the prototype was updated to incorporate feedback from the research activity:

1. A workshop with therapists (n=4) to provide feedback (see Table 2) on the website prototypes developed from the service user and carer feedback.
2. Individual discussions with 2 different therapists from those in the workshops who provided feedback during the design process.
3. A mixed stakeholder workshop consisting of service users, carers, and therapists who participated in stages 1 and 2, as well as new participants, was conducted for feedback on the finalized prototype (Table 3).

**Table 2.** Workshop 2 design changes—therapist feedback (Alpha phase).

Feedback item	Design/software modification	Project solution
(1) Accessibility		
The navigation of the site must be very simple for service users and for therapists.	A simple link to navigate to relevant webpages was developed. This removed the need for service users to navigate large sections of the website, as the link directed them to the specific content designed for them at that stage in the therapeutic process.	Service users received the individual links by email. Therapists were provided with a “Copy Link” button to enable them to paste the link into an email.
Embed training in the website for therapists.	Training video created for therapists. Development of a “Help Page.”	“Help Page” and training video included on the website.
Some service users will not have access to digital devices or data plans to access CaFI^a^:Digital.	No action required.	Tablets purchased and provided to service users where needed. The study purchased mobile data plans to ensure the website could be accessed.
Service users will also require printed materials to ensure equity of access to those who prefer to work with hard-copy materials.	No action required.	Printed materials were prepared for use with all service users.
(2) Positive feedback		
Participants felt the site looked very good.	No action required.	No action required.

a CaFI: culturally adapted family intervention.

**Table 3.** Workshop 3 mixed stakeholder feedback—Alpha phase (Consensus).

Feedback item	Design/software modification	Project solution
(1) Positive feedback		
Participants felt the site was visually appealing.	No action required.	No action required.
Participants felt the site looked very easy to use for everyone.	No action required.	No action required.
Participants felt the site looked very good.	No action required.	No action required.
(2) Accessibility		
Queries about whether the website worked across different kinds of devices (tablets, mobile, desktop).	Confirmed that the website worked across multiple devices.	No action required.

**Figure 1.**
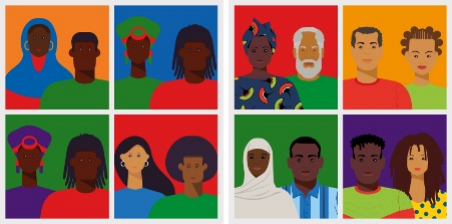
Four images of people from the original prototype (left), and four updated images (right). The updated images included people from North African communities, incorporating a variety of skin tones, as well as individuals wearing culturally significant clothing, such as hijabs.

**Figure 2.**
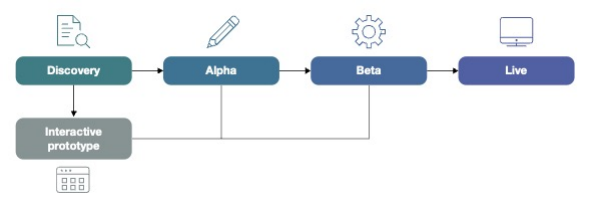
Flowchart showing the development stages of the interactive prototype.

The beta-test phase involved usability testing with 9 participants (including service users, carers, therapists, researchers, and software team staff) to ensure the final website, co-designed through the Discovery and Alpha phases, was defect-free and ready for use. The beta-test phase was completed by sending email requests to service users from the NHS and voluntary sectors and therapists with details of exercises to complete in their own time (for details of the tasks, see Multimedia Appendix 1). The tasks were designed to provide feedback to the team on the real-world usability and accessibility of the platform. Each task was designed to last roughly 1 hour. Figure 2 shows the development stages of the prototype.

### Accessibility

The website prototypes shown in the workshops had been designed and tested to be Web Content Accessibility Guidelines 2.1 compliant [28] as a baseline approach to accessibility. An accessibility discussion was included in the focus group to identify and review the specific accessibility needs participants might experience when using the website. Participants were specifically asked whether they anticipated encountering any barriers to accessing or using the website and which devices they would find most useful for accessing the website. The focus of accessibility discussions in the workshops was to identify specific additional accessibility needs that may have been particularly relevant for this target population.

### MoSCoW Prioritization

Aligned with Agile approaches [26], we adopted the MoSCoW (Must have, Should have, Could have, Won’t have) prioritization system [29] to triage requirements. The MoSCoW approach is widely used in software development to determine the most important features and deliverables across mixed stakeholder groups. In the MoSCoW approach, design features of the website were ranked as “must have,” “should have,” “could have,” and “won’t have.” Features that were ranked as “must have” were considered essential by stakeholders; “should have” features were deemed important but would not affect the functionality of the website if they were not included; “could have” features were not necessarily related to the key aims of CaFI:Digital but were seen as additional desirable features; and features classified as “won’t have” were either not priorities for stakeholders or not feasible to deliver within the scope of this study. Workshop facilitators supported discussion when different priorities were identified, aiming to arrive at a consensus decision. Data from the workshops were then analyzed by the project research associate, project technical team lead, and UX designer. Notes were taken during the workshops by the project research associate. Following the workshops, the notes were discussed in detail by the project team and developed into itemized requirements lists for prioritization and implementation in the software. Similar to our approach taken in other co-design projects [30], the technical team analyzed the workshop notes, identifying common requirements and themes in the data. They grouped key themes from the notes after each workshop and identified their technical feasibility. Key requirements were then circulated to the wider research team for review. The technical team and research team then met to review and discuss the workshop insights collectively. The agreed key requirements were then prioritized for technical implementation by the software team. Where decisions were outstanding after the workshops, the research team and technical teams made the final decisions, guided by the necessity to deliver the project on time and within budget.

### Tracking Usage and Engagement Metrics

Usage and engagement analytics were embedded within the platform following the AMUsED (Analyzing and Measuring Usage and Engagement Data) framework for digital health interventions [31]. The main goal was to track analytics within the platform that could help in understanding which elements of content were most visited by participants.

## Results

The co-design workshops generated user needs-led software design requirements and modifications to prototypes that were demonstrated by the project UX designer. A summary of requirements and changes is included in Tables1^3^.

### Workshop Feedback

Feedback in the service user and carer workshops (Table 1) focused on the need to ensure that the website was easy to use and simple to navigate. An emphasis was placed on accessibility: making the font size large to support people with visual impairments, making the text easy to read, and providing adequate support and training for people unfamiliar with digital technologies. Diversity in the images portrayed on the website was also requested, particularly in terms of diversity of skin tones depicted.

The main themes from the first workshop with service users and carers (Table 1) related to (1) the accessibility of the website in terms of navigation, disability, digital literacy, and lack of hardware; (2) the content of the website being diverse and representative of people of sub-Saharan African and Caribbean descent; and (3) positive feedback in terms of ease of use of the website and the look and feel of the content. It is important to note that discussions relating to accessibility also raised the possibility of providing audio versions of all materials; however, this was not supported in this project due to time and cost limitations. Each of the feedback items was agreed by a majority within the workshop group as a need or priority for the software.

The main themes from the second workshop with therapists (Table 2) related to (1) the accessibility of the website in terms of navigation, training therapists to use the content, lack of hardware, and a preference from some service users to work with physical materials and (2) the website being visually appealing to therapists.

The main themes from the mixed stakeholder workshop related to (1) the website being visually appealing and (2) accessibility of the website across different devices (ie, phones and laptops).

Feedback in the service user and carer workshops (Table 1) focused on the need to ensure that the website was easy to use and simple to navigate. An emphasis was placed on accessibility: making the font size large to support people with visual impairments, making the text easy to read, and providing adequate support and training for people unfamiliar with digital technologies. Diversity in the images portrayed on the website was also requested, particularly in terms of diversity of skin tones depicted.

Feedback in the therapist workshops included recommendations to prioritize simplicity of design; ensure ease of site navigation; embed training within the site; and ensure supplementary printed materials were provided to participants. The service user and carer workshop identified the positives of using digital technology to supplement therapy delivery, including reporting how digital technology “offered a way to engage with the world” even when people felt very unwell. Poor accessibility of some digital platforms was raised as a concern by service user participants in the workshops, several of whom mentioned the need to ensure the website worked well for those with visual impairments.

### Analyzing and Prioritizing Workshop Feedback

Requirements from the workshops were then actioned by the software team or wider research team, as appropriate. The aim was to implement as many requirements as possible within the time and budget available. A small number of items were deemed “out of scope” because of the time and cost implications of implementation, detailed in Tables1^3^.

### Digital Inequities

Participants across all workshops expressed concern about people who had limited or no access to digital technology and/or had poor digital literacy skills, stressing the need for equity in the delivery of digital solutions and therapeutic interventions more widely.

### Test Results

After all the changes from the co-design workshops had been addressed, as described in Tables1^3^, 9 volunteers were asked to test the website on their own devices. The purpose of this phase was to identify any critical or major technical defects with the website that needed to be resolved. Volunteers represented (1) service users and families, (2) therapists, and (3) networks of the research team, including people with therapeutic, clinical, and/or digital backgrounds. A major problem was reported on a specific version of the iPhone, which blocked users from logging in. This was resolved by the software team, and the website was retested on this iPhone version. No other critical or major defects were reported by beta testers. Positive feedback on the beta testing is reported in the table below.

### Accessibility-Related Changes

Accessibility was identified as a key area of focus throughout the co-design and development work packages. Additional accessibility needs were identified through the workshops. In the co-design workshops, visual impairment was identified as a common problem among the target population. To respond to this, a larger-than-usual font size was used as the default, and training was included with the trial rollout to advise how to increase the font of the website on common web browsers. Color contrast was also assessed to ensure the website worked well for people with visual impairments, a point raised by service users in Workshop 1 and by the wider research team in team meetings.

## Discussion

### Principal Results

To our knowledge, this is one of few studies to co-design a culturally informed digital mental health intervention to address the disparities in psychosis care experienced by people of sub-Saharan African and Caribbean descent. Another example of this is from the Care Pathways therapeutic intervention in America, which, like CaFI:Digital, was co-designed with Black American women and mental health professionals [32]. Unlike CaFI:Digital, the intervention in America was a mindfulness-based application to support anxiety and depression [32]. The CaFI:Digital co-design activities ensured the priorities of the end users of the platform are met. The target population for the CaFI trial has historically been underserved digitally through lack of provision of digital mental health tools as well as in terms of access to evidence-based psychological interventions. CaFI:Digital is an attempt to redress this imbalance. The CaFI:Digital intervention will be evaluated as part of the process evaluation of the randomized controlled trial. Metrics that include login frequency, duration, module completion, engagement with materials, accessibility, and user-friendliness will be used to evaluate usage and engagement.

The CaFI trial offered an opportunity to develop and evaluate a digital mental health intervention that was co-designed. Digitization also provides an opportunity to engage family members in therapy who may live far away from each other. This is particularly relevant for the CaFI trial target population, many of whom have family members located in different locations around the world. Moreover, unlike face-to-face interventions, the CaFI:Digital platform can be readily adapted and scaled for worldwide delivery. This aligns with national and international policy and practice to address ethnically based inequalities, aligning with both NHS priorities in the United Kingdom [33] and global approaches to enhancing delivery of health care using digital tools [34].

The findings of this study are consistent with recent policy shifts in the United Kingdom, notably the NHS Long Term Plan (2019) and NHS England’s Inclusive Digital Healthcare Framework (2023), which advocate for the increased use of digital tools to enhance accessibility and equity in health care delivery [35,36]. Furthermore, CaFI:Digital aligns with NHS inclusive research practice foregrounding cultural competence, stakeholder engagement, and the reduction in health inequalities [37]. The co-design approach used in the platform’s development reflects the NHS commitment to involving diverse, underrepresented communities in research and developing interventions tailored to meet the needs of Black, Mixed heritage, and other racially minoritized populations.

The combination of co-design and Agile software approaches used for CaFI:Digital enabled the software team to identify and respond to requirements and refinements identified by service users, their families and carers, and the therapists, despite the constraints on face-to-face meetings during the COVID-19 pandemic. The use of the MoSCoW prioritization system supported the rapid development cycle required to respond to the pandemic and stakeholder needs. This was particularly helpful when time-consuming or resource-intensive recommendations were identified. A key concern for participants during the development phase of the website was ensuring the website was simple to use and accessible to all, and dedicated workstreams were developed to address specific accessibility concerns raised. The beta test ensured that the software was defect-free and ready to use in the live trial. Inevitably, more suggestions were made at the co-design workshops than there was time or budget to implement. Future versions of the software could seek to address these recommendations (eg, by providing audio versions of all the website materials).

At the time of writing, the CaFI:Digital is being used in a live trial, so it is not possible to report trial outcomes or to present analyzed data on usage and engagement. We are therefore unable to comment on real-world impact and effectiveness. Another limitation is that not all suggested features, such as providing audio versions of all materials, were included because of resource constraints. Finally, although the platform has been co-designed to meet accessibility needs in terms of simplicity, conveying information within the CaFI:Digital platform can also depend on the competency of the therapist. The contribution of the CaFI trial to evidence-based practice will be evaluated as part of the ongoing trial.

The co-design and development phases have sought to make the website as usable and accessible as possible by working with service users, their families, and therapists throughout the co-design process. CaFI:Digital provides access to psychological therapies for people in underrepresented populations. Analysis from the CaFI trial using the engagement analytics embedded within the website combined with qualitative research on user experiences of the platform will enrich our understanding of how participants experienced the CaFI:Digital platform during the trial. Evaluating online group therapy interventions with underserved populations will enhance our understanding of how technology enhances care or perpetuates inequalities for already underserved and “seldom heard” populations.

The CaFI:Digital platform presents unique opportunities to respond to NICE recommended policy guidelines in the United Kingdom [13] by enabling remotely delivered, culturally adapted family therapy. Policy recommendations stemming from this research should prioritize the integration of digital health solutions within national and international health care frameworks, particularly in relation to ethnic minority groups who experience disproportionately high rates of serious mental illnesses such as schizophrenia and related psychoses and systemic health care exclusion.

### Conclusions

CaFI:Digital, an evidence-based digital therapy platform, aspires to be at the forefront of efforts to address the gap in access to mental health care for UK Black and other minoritized communities, working toward conforming to the NICE evidence standards framework for health technologies. There is potential for the platform to be rolled out internationally for people of sub-Saharan and Caribbean descent, at scale and at a competitive cost, to provide access to treatment to historically excluded and underserved groups. As with all digital mental health interventions, the potential for CaFI:Digital to widen inequalities through “digital exclusion” (by further restricting access to treatment only to people who have capabilities to use digital tools) must also be considered. It is therefore important to situate CaFI:Digital within a landscape of options for the target population rather than to promote it as a single solution that will be relevant for everyone.

However, CaFI:Digital also has the potential to address longstanding digital inequalities, aligned with 2023 NHS England guidance [35]. The NHS England guidance outlines ways to make digital health care more accessible by maximizing the relatability of the technologies, conducting mixed methods research in the digital space, and actioning user feedback, many of which have been addressed in the development of CaFI:Digital. Moreover, for a population who often have family members located internationally, the remote therapeutic delivery enables the inclusion of family members previously excluded. This is important given the disproportionate rates of diagnosis and lack of access to psychological therapies experienced by this population. Our hope is that CaFI:Digital can begin to address this historic gap in provision of CaFIs for Black populations.

## Supplementary material

10.2196/73246 Multimedia Appendix 1 Beta testing—therapist.

## Abbreviations

AMUsED: Analyzing and Measuring Usage and Engagement Data
CaFI: Culturally-adapted Family Intervention
MoSCoW: Must have, Should have, Could have, Won’t have
NHS: National Health Service
NICE: National Institute for Health and Care Excellence

## References

[1] Schofield P, Kordowicz M, Pennycooke E, Armstrong D (2019). Ethnic differences in psychosis-Lay epidemiology explanations. Health Expect.

[2] Fearon P, Kirkbride JB, Morgan C (2006). Incidence of schizophrenia and other psychoses in ethnic minority groups: results from the MRC AESOP Study. Psychol Med.

[3] Morgan C, Fearon P, Lappin J (2017). Ethnicity and long-term course and outcome of psychotic disorders in a UK sample: the ÆSOP-10 study. Br J Psychiatry.

[4] Pilling S, Bebbington P, Kuipers E (2002). Psychological treatments in schizophrenia: I. Meta-analysis of family intervention and cognitive behaviour therapy. Psychol Med.

[5] Camacho-Gomez M, Castellvi P (2020). Effectiveness of family intervention for preventing relapse in first-episode psychosis until 24 months of follow-up: a systematic review with meta-analysis of randomized controlled trials. Schizophr Bull.

[6] Ellis BH, Winer JP, Murray K, Barrett C (2019). The Massachusetts General Hospital Textbook on Diversity and Cultural Sensitivity in Mental Health.

[7] Snider TC, Raglin Bignall WJ, Hostutler CA, Hoet AC, Walker BL, Bailey J (2020). Development and implementation of a culturally tailored early childhood program in an integrated pediatric primary care practice. Clin Pract Pediatr Psychol.

[8] Edge D, Grey P (2018). An assets-based approach to co-producing a culturally adapted family intervention (CaFI) with African Caribbeans diagnosed with schizophrenia and their families. Ethn Dis.

[9] Devonport TJ, Ward G, Morrissey H (2023). A systematic review of inequalities in the mental health experiences of Black African, Black Caribbean and Black-mixed UK populations: implications for action. J Racial Ethn Health Disparities.

[10] Jensen E, Carr R, Degnan A, Berry K, Edge D (2021). Exploring service user and family perspectives of a culturally adapted family intervention (CaFI) for African-Caribbean people with psychosis: a qualitative study. Br J Clin Psychol.

[11] Morgan C, Dazzan P, Morgan K (2006). First episode psychosis and ethnicity: initial findings from the AESOP study. World Psychiatry.

[12] (2020). Why have Black and South Asian people been hit hardest by COVID-19. Office for National Statistics (ONS).

[13] (2014). Psychosis and schizophrenia in adults: prevention and management, section 1.1.2 race, culture and ethnicity. https://www.nice.org.uk/guidance/cg178/resources/psychosis-and-schizophrenia-in-adults-prevention-and-management-pdf-35109758952133.

[14] (2023). Health and social care delivery research (HSDR) programme. National Institute for Health and Care Research (NIHR).

[15] Edge D, Degnan A, Cotterill S (2016). Culturally-adapted family intervention (CaFI) for African-Caribbeans diagnosed with schizophrenia and their families: a feasibility study protocol of implementation and acceptability. Pilot Feasibility Stud.

[16] Edge D, Degnan A, Cotterill S (2018). Culturally adapted Family Intervention (CaFI) for African-Caribbean people diagnosed with schizophrenia and their families: a mixed-methods feasibility study of development, implementation and acceptability. Health Serv Deliv Res.

[17] Pan D, Sze S, Minhas JS (2020). The impact of ethnicity on clinical outcomes in COVID-19: a systematic review. EClinicalMedicine.

[18] Masters JR (2002). HeLa cells 50 years on: the good, the bad and the ugly. Nat Rev Cancer.

[19] Thomas SB, Quinn SC (1991). The Tuskegee Syphilis Study, 1932 to 1972: implications for HIV education and AIDS risk education programs in the Black community. Am J Public Health.

[20] Nuriddin A, Mooney G, White AIR (2020). Reckoning with histories of medical racism and violence in the USA. Lancet.

[21] Malterud K, Siersma VD, Guassora AD (2016). Sample size in qualitative interview studies: guided by information power. Qual Health Res.

[22] LaDonna KA, Artino AR, Balmer DF (2021). Beyond the guise of saturation: rigor and qualitative interview data. J Grad Med Educ.

[23] Adobe XD Platform.

[24] Marvel.

[25] Maze.

[26] Dingsøyr T, Nerur S, Balijepally V, Moe NB (2012). A decade of agile methodologies: towards explaining agile software development. J Syst Softw.

[27] (2023). Agile delivery. GOV.UK.

[28] Web content accessibility guidelines (WCAG) 2.1. World Wide Web Consortium (W3C).

[29] Wiegers KE, Beatty J (2013). Software Requirements.

[30] Sharma V, McDermott J, Keen J, Foster S, Whelan P, Newman W (2024). Pharmacogenetics clinical decision support systems for primary care in England: co-design study. J Med Internet Res.

[31] Miller S, Ainsworth B, Yardley L (2019). A framework for Analyzing and Measuring Usage and Engagement Data (AMUsED) in digital interventions: viewpoint. J Med Internet Res.

[32] O’Leary TK, Batti V, Dhindsa S (2025). “It was empowering”: care pathways a novel culturally informed inspiration card game for community-engaged co-design with Black American women and clinicians.

[33] (2023). 2023/24 priorities and operational planning guidance. https://www.england.nhs.uk/wp-content/uploads/2022/12/PRN00021-23-24-priorities-and-operational-planning-guidance-v1.1.pdf.

[34] (2021). Global strategy on digital health 2020–2025. https://iris.who.int/server/api/core/bitstreams/1f4d4a08-b20d-4c36-9148-a59429ac3477/content.

[35] (2023). Inclusive digital healthcare: a framework for NHS action on digital inclusion. National Health Service England.

[36] (2019). The NHS long term plan. https://webarchive.nationalarchives.gov.uk/ukgwa/20250707103631/https://www.longtermplan.nhs.uk/publication/nhs-long-term-plan/.

[37] (2023). A national framework for NHS – action on inclusion health. National Health Service England.

