# Supplementary material for: Co-Designing a Digital Platform to Support a Culturally Adapted Family Intervention (CaFI:Digital) for Psychosis Among People of Sub-Saharan African and Caribbean Descent: Agile Co-Design Approach
Source: JMIR Form Res. 2026 Mar 25;10:e73246. doi: 10.2196/73246 (PMC13015912; doi:10.2196/73246)
Supplement: Multimedia Appendix 1 [file formative-v10-e73246-s001.docx]

**Appendix 1**

**Beta Testing – Therapist**

**Exercise:**

1. Login to [[Cafi](http://www.cafi-study.org) URL provided] with the username and password provided.
2. Add participant and note the username and password
3. Open the participant content and browse the 5 components. You do not need to review the content in details. The goal is ensure you can see and navigate the content and that it looks clear and accessible.
4. Go to Component 2 and find the content 'Schizophrenia and psychosis', and click the 'share' link to copy the url to your clipboard. Try and paste the link into a separate page (e.g. email) and ensure it directs to the correct page.
5. Return back to participant control panel, click on participant to view their activity.

**Questions:**

1. Was the login process clear and straightforward?
2. Were you able to create a participant?
3. Do you have any general comments about the participant content?
4. Could you find the ‘Schizophrenia and psychosis’ link? Were you able to copy the URL?
5. Were you able to view participant activity?
6. Any general comments on the CaFI:Digital website?

**Beta Testing – Service User**

We would ask that you complete the exercise below and then reply by email to the questions.  We anticipate this taking no longer than 1 hour.  The purpose of this exercise is for us to check that the website will be accessible to participants in the project.

**Exercise:**

1. Navigate to this URL [[](https://www.cafi-study.org/)CaFI URL provided]
2. Log in using the details below
3. Return to this email and select a link from the below.  Visit the links below [2 links to Cafi content links provided]. You do not need to read the content in detail.
4. You should only be able to view the content relevant to the link; there will be limited options to navigate around the site.
5. Note: Only do step 5 if you are using a computer (PC, laptop). Follow this link [Cafi worksheet content link provided] and download the worksheet.
6. If you downloaded the worksheet in Step 5, you can now delete it from your computer.

**Questions:**

- What type of device were you using? (e.g. Samsung smartphone, laptop, ipad)
- Was the login process clear and straightforward?
- Could you view the content from the link?
- Could you download the worksheet?
- Do you have any general comments on the CaFI:Digital website?
